# Effect of opposing needling on motor cortex excitability in healthy participants and in patients with post-stroke hemiplegia: study protocol for a single-blind, randomised controlled trial

**DOI:** 10.1186/s13063-021-05443-x

**Published:** 2021-07-22

**Authors:** Mindong Xu, Yinyu Zi, Jianlu Wu, Nenggui Xu, Liming Lu, Jiahui Liu, Yanling Yu, Haofeng Mo, Weifeng Wen, Xiaorong Tang, Wenjuan Fan, Yu Zhang, Churong Liu, Wei Yi, Lin Wang

**Affiliations:** 1 Medical College of Acu-Moxi and Rehabilitation, Guangzhou University of Chinese Medicine, Guangzhou, 510000 China; 2 South China Research Center for Acupuncture and Moxibustion, Medical College of Acu-Moxi and Rehabilitation, Guangzhou University of Chinese Medicine, Guangzhou, 510000 China; 3 College of Chinese Medicine, Guangzhou University of Chinese Medicine, Guangzhou, 510000 China; 4 Rehabilitation Department, Guangdong 999 Brain Hospital, Guangzhou, 510000 China; 5 College of Health Medicine, Chongqing Youth Vocational and Technical College, Chongqing, 400712 China; 6 Massage Therapy Center, The First Affiliated Hospital of Anhui University of Chinese Medicine, Hefei, 230012 China

**Keywords:** Opposing needling, Stroke, Post-stroke hemiplegia, Transcranial magnetic stimulation, Resting motor threshold, Motor-evoked potential

## Abstract

**Background:**

Opposing needling has an obvious curative effect in the treatment of post-stroke hemiplegia; however, the mechanism of the opposing needling in the treatment of post-stroke hemiplegia is still not clear. The purpose of this study is to investigate the effect of opposing needling on the excitability of primary motor cortex (M1) of healthy participants and patients with post-stroke hemiplegia, which may provide insight into the mechanisms of opposing needling in treating post-stroke hemiplegia.

**Methods:**

This will be a single-blind, randomised, sham-controlled trial in which 80 healthy participants and 40 patients with post-stroke hemiplegia will be recruited. Healthy participants will be randomised 1:1:1:1 to the 2-Hz, 50-Hz, 100-Hz, and sham electroacupuncture groups. Patients with post-stroke hemiplegia will be randomised 1:1 to the opposing needling or conventional treatment groups. The M1 will be located in all groups by using neuroimaging-based navigation. The stimulator coil of transcranial magnetic stimulation (TMS) will be moved over the left and right M1 in order to identify the TMS hotspot, followed by a recording of resting motor thresholds (RMTs) and motor-evoked potentials (MEPs) of the thenar muscles induced by TMS before and after the intervention. The primary outcome measure will be the percent change in the RMTs of the thenar muscles at baseline and after the intervention. The secondary outcome measures will be the amplitude (μV) and latency (ms) of the MEPs of the thenar muscles at baseline and after the intervention.

**Discussion:**

The aim of this trial is to explore the effect of opposing needling on the excitability of M1 of healthy participants and patients with post-stroke hemiplegia.

**Trial registration:**

Chinese Clinical Trial Registry ChiCTR1900028138. Registered on 13 December 2019.

**Supplementary Information:**

The online version contains supplementary material available at 10.1186/s13063-021-05443-x.

## Background

According to World Health Organization statistics, stroke is the second highest cause of death and a leading cause of disability worldwide [1]. In the USA, stroke is the fourth position in the leading causes of mortality [2] and a leading cause of serious long-term disability [3]. The total direct annual stroke-associated medical expenses are expected to increase from $71.55 billion to $183.13 billion between 2012 and 2030 [4]. In Australia, there are 27,400 new cases of stroke in 2020. The economic impact of stroke is staggering, causing direct economic costs of $6.2 billion, and premature deaths and welfare losses of $26 billion [5]. In China, according to the Chinese stroke epidemiology survey published in the journal *Circulation* in 2017, the incidence and mortality rates associated with stroke were 246.8/100 000 and 114.8/100 000 person-years, representing approximately 2.4 million new cases each year and approximately 1.1 million deaths each year. Among incident and prevalent strokes, ischemic stroke constituted 69.6% and 77.8% of the cases, respectively [6]. Thus, stroke shows the characteristics of high incidence, high morbidity, and high disability [7–9].

The most effective evidence-based treatment for ischemic stroke is tissue plasminogen activator (tPA) administration within 4.5 h or mechanical thrombectomy (MT) within 6 h, which can salvage the tissue at risk in the penumbra of a brain infarct and reduce further disability if administered promptly after stroke onset [10, 11]. A retrospective cohort study suggested that a shorter time to treatment with tPA was associated with lower all-cause mortality and lower all-cause readmission at 1 year [12]. However, in China, only about 10–20% of ischemic stroke patients arrive at hospitals within 3 h, and less than 3% of stroke patients are treated with tPA [13]. Most ischemic stroke patients are not treated in time, which delays the therapeutic effects, leading to higher mortality and disability rates. For patients with stroke at the convalescence stage, the existing treatments mainly include conventional antiplatelet therapy (monotherapy or combinations of aspirin, clopidogrel, and cilostazol) [14], complementary and alternative medicines (herbal medicine, Chinese conventional exercise therapy, acupuncture, moxibustion, etc.) [15–19], and rehabilitation therapy [20]. Most patients in China with post-stroke hemiplegia are treated with a combination of Chinese and modern medicine. One study recommended acupuncture for patients with various stroke recovery periods [21]. Acupuncture has been used to treat post-stroke hemiplegia for more than 2000 years and has shown significant therapeutic effects [22, 23]. Opposing needling, one of the most common acupuncture treatments for post-stroke hemiplegia, comprising the selection of acupuncture points contralateral to the diseased side [24]. A systematic review suggested that in comparison with ipsilateral acupuncture, opposing needling showed a superior effect on the recovery rate and influence the recovery from post-stroke hemiplegia by improving the motor function of the affected limbs, the daily living ability of the patients, and even neurological deficits [25]. Although opposing needling has obvious curative effects in the treatment of post-stroke hemiplegia, the mechanisms underlying its therapeutic effects remain unclear. The existing research on the mechanisms of opposing needling mainly involved experimental animals, and only a few studies have assessed patients with post-stroke hemiplegia. Thus, with a single-blind, randomised controlled design, this study aims to investigate the effect of electroacupuncture and opposing needling on the excitability of the M1 under physiological and pathological conditions.

## Objectives

The primary objective is to investigate the effect of electroacupuncture on the excitability of the M1 in healthy participants, and then to further investigate the effect of opposing needling on the M1 excitability of patients with post-stroke hemiplegia. Secondary objectives are to explore the optimal frequency of electroacupuncture for motor cortex excitability in healthy participants and explore a potential approach to predict the possibility of recovery in patients with post-stroke hemiplegia.

## Methods/design

### Study design

**Fig. 1:**
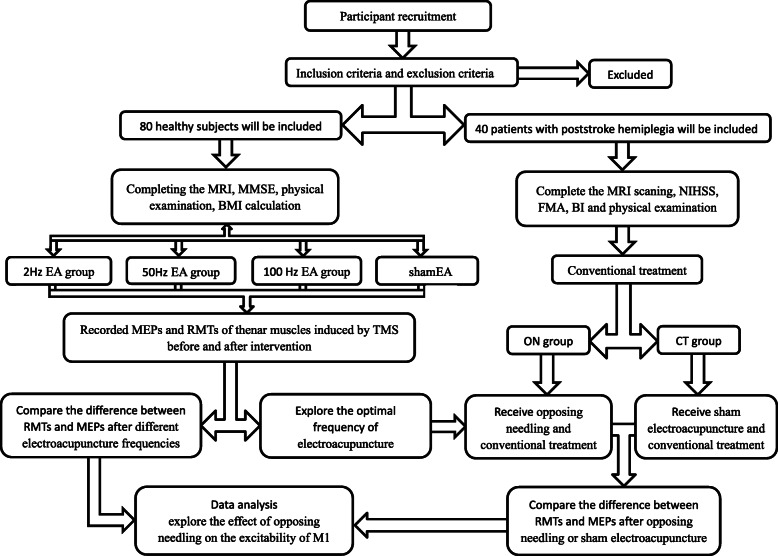
Consort flow chart. MMSE, Mini-Mental State Examination; BMI, body mass index; MRI, magnetic resonance imaging; NIHSS, National Institutes of Health stroke scale; FMA, Fugl-Meyer assessment; BI, Barthel index; EA, electroacupuncture; MEP, motor-evoked potential; RMT, resting motor threshold; TMS, transcranial magnetic stimulation; ON, opposing needling; CT, conventional treatment

**Fig. 2:**
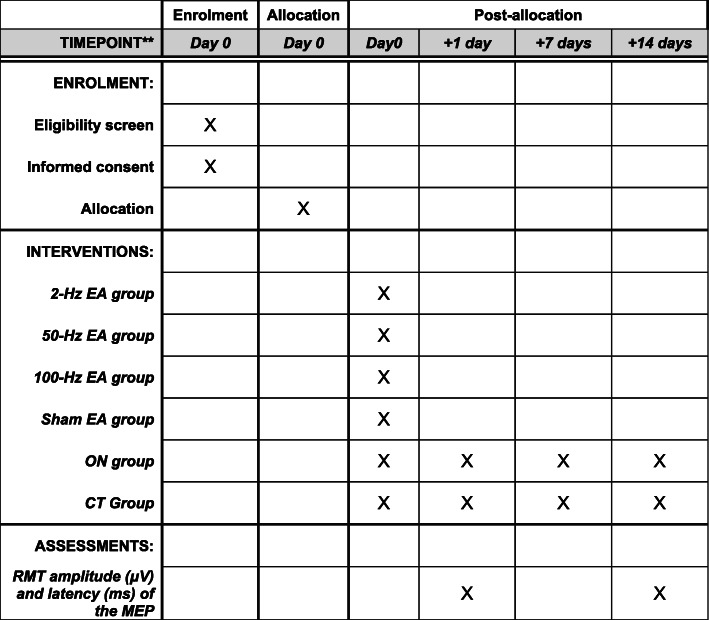
Schematic overview of enrolment, intervention, and assessments

This will be a single-blind, randomised, sham-controlled trial (RCT) conducted in accordance with the Declaration of Helsinki. This RCT will be conducted at the South China Research Center for Acupuncture and Moxibustion, Guangzhou University of Chinese Medicine and Guangdong 999 Brain Hospital from January 2020 to January 2022. All participants will provide signed informed consent before proceeding with the trial. All study protocols have been reviewed and approved by the China Ethics Committee of Registering Clinical Trials (ChiECRCT20190223). This study has been registered with the Chinese Clinical Trial Registry (ChiCTR1900028138). The protocol follows the Standard Protocol Items: Recommendations for Interventional Trials (SPIRIT) 2013 Statement. The SPIRIT checklist is provided as Additional file 1. The study design is presented in the flowchart in Figs. 1 and 2.

### Recruitment

Recruitment for this RCT will be initiated by posting study recruitment flyers at the Guangzhou University of Chinese Medicine and Guangdong 999 Brain Hospital. Interested patients will be instructed to contact the recruitment staff by telephone or email and then requested to provide consent before participation in the study. Recruitment staff will be responsible for providing details regarding this RCT to the patients and enrolling participants for the study. Healthy individuals who meet the study criteria will be invited to the South China Research Center for Acupuncture and Moxibustion, while patients with post-stroke hemiplegia who meet the enrolment criteria will be invited to the Guangdong 999 Brain Hospital to participate in the study.

### Inclusion criteria

Healthy individuals who meet the following inclusion criteria will be eligible to participate in this study: (1) age between 18 and 65 years, male or female sex; (2) good health and no history of stroke; (3) normal physical examination results; (4) normal results on the Mini-Mental State Examination (MMSE; a measure of cognitive impairment); (5) a body mass index (BMI) between 18.5 and 24 kg/m^2^; and (6) willingness to sign the informed consent form.

The inclusion criteria for patients with post-stroke hemiplegia will be as follows: (1) conformance with the diagnostic criteria proposed in the *Guidelines for Diagnosis and Treatment of Common Internal Diseases in Chinese Medicine Diseases of Modern Medicine* (ZYYXH/T19-2008) and the *Guidelines for the diagnosis and treatment of acute ischemic stroke in China 2018*; (2) stroke history of no more than 6 months; (3) age between 18 and 75 years, with no limitations related to sex; (4) Fugl-Meyer assessment (FMA) score less than 95 points for the right limb, muscle strength of the right limb less than Grade 4, and FMA score and muscle strength of the left limb within the normal range; (5) consent to undergo acupuncture; (6) adequate consciousness, stable vital signs, no obvious mental retardation, no obvious obstacles in hearing, MMSE score within the normal range (normal thresholds: illiteracy, > 17 points; elementary school, > 20 points; junior high school and above, > 24 points), and ability to undergo basic procedures such as rehabilitation training; and (7) willingness to participate in the study and sign the informed consent form.

### Exclusion criteria

Healthy participants with any of the following conditions will be excluded: (1) use of a cardiac pacemaker or implanted metal (such as brain metal electrodes [except titanium], cochlear implants, or pulse generators); (2) conditions that may potentiate seizures (such as a history of epilepsy, sleep deprivation, alcohol dependence, or ingestion of olanzapine or lithium carbonate); and (3) pregnancy or lactation.

Patients who meet any of the following conditions will be excluded: (1) patients who have received other treatments that may affect the observations in this study and those who do not follow the study protocol; (2) patients with severe heart, liver, spleen, lung, or kidney disease or mental illness; (3) patients with aphasia, deafness, severe cognitive impairment, or other conditions that hinder their ability to communicate properly; (4) patients unwilling to undergo acupuncture or basic therapy; (5) patients who cannot complete the treatment and may not show good compliance; (6) patients who did not experience physical paralysis after illness; (7) patients who experienced stroke more than three times; (8) patients who received a cardiac pacemaker or other implanted devices such as brain metal electrodes (except titanium), cochlear implants, or pulse generators; (9) patients with conditions that may potentiate seizures, such as a history of epilepsy, sleep deprivation, alcohol dependence, or ingestion of olanzapine or lithium carbonate; and (10) pregnant or lactating patients.

### Randomisation and allocation concealment

A total of 80 healthy participants and 40 patients with post-stroke hemiplegia will be recruited. The 80 healthy participants will be randomised 1:1:1:1 to 2-Hz, 50-Hz, 100-Hz, and sham electroacupuncture groups. The 40 patients with post-stroke hemiplegia will be randomised 1:1 to an opposing needling group or a conventional treatment group. Randomisation will be performed by the Package for Encyclopedia of Medical Statistics 3.1 software (West China School of Public Health, Sichuan, China) and will be overseen by an independent statistician. Healthy participants will be sequentially assigned a random number from 1 to 80, and patients with post-stroke hemiplegia will be sequentially assigned a random number from 1 to 40. Opaque sealed envelopes will be numbered consecutively with a serial number on the outside for randomisation and will contain the allocation information. The envelope will be opened when the participant or patient enters the trial after providing informed consent. Healthy participants will then be allocated to one of the four groups and the patients will be allocated to one of the two groups. All participants and patients will receive an intervention according to their group allocation. The random allocation sequence and the opaque sealed envelopes will be kept by two independent researchers.

### Blinding

Given the nature of the interventions, blinding of the acupuncturists to the trial condition will be difficult. Therefore, in order to maintain blinding, the participants, researchers and the statistician will be blinded by the randomisation procedure. The data will be analysed by two statisticians who will otherwise not be involved in this trial and will remain naive to all study processes.

### Study procedure

The healthy volunteers who consent to participate in the study will complete magnetic resonance imaging (MRI) in Guangdong 999 Brain Hospital, and finish the MMSE, physical examination, and BMI evaluation in an examination room at South China Research Center for Acupuncture and Moxibustion, Medical College of Acu-Moxi and Rehabilitation, Guangzhou University of Chinese Medicine. The patients with post-stroke hemiplegia who consent to participate in the study will undergo MRI, National Institutes of Health Stroke Scale (NIHSS), FMA, Barthel index, and physical examination at a rehabilitation room in Guangdong 999 Brain Hospital. All participants will sit on a treatment chair facing the neuroimaging navigation and TMS machine to enable the location of the correct brain regions.

This study will be divided into the following parts:

Part 1: Effect of different frequencies of electroacupuncture on the excitability of M1 in healthy participants. Firstly, the cortical stimulation will be performed on the left and right M1 of all healthy participants while recording the RMTs and MEPs of bilateral thenar muscles. And then, they will receive 2-Hz, 50-Hz, 100-Hz, or sham electroacupuncture intervention on their left limbs for 20 minutes to explore the optimal frequency. Finally, the RMTs and MEPs both will be tested immediately after intervention within 5 minutes again.

Part 2: Effect of opposing needling on the excitability of M1 in patients with post-stroke hemiplegia. Firstly, the cortical stimulation will be performed on the left and right M1 of patients with post-stroke hemiplegia before the treatments while recording the RMTs and MEPs of bilateral thenar muscles. And then, on the premise of the same conventional treatments and rehabilitation trainings, all the patients will opposing needling as the optimal frequency of electroacupuncture acquired from Part 1 or sham electroacupuncture. After ten opposing needling treatments or ten sham electroacupuncture interventions, the RMTs and MEPs both will be detected again.

Part 3: The physiological and pathological data will be combined to determine the relationship between M1 excitability and stroke rehabilitation, explore the effect of opposing needling on M1 excitability, propose a possible hypothesis for the treatment of post-stroke hemiplegia by opposing needling, and explore the possibility of using M1 excitability to predict stroke recovery.

### Neuroimaging navigation

**Fig. 3:**
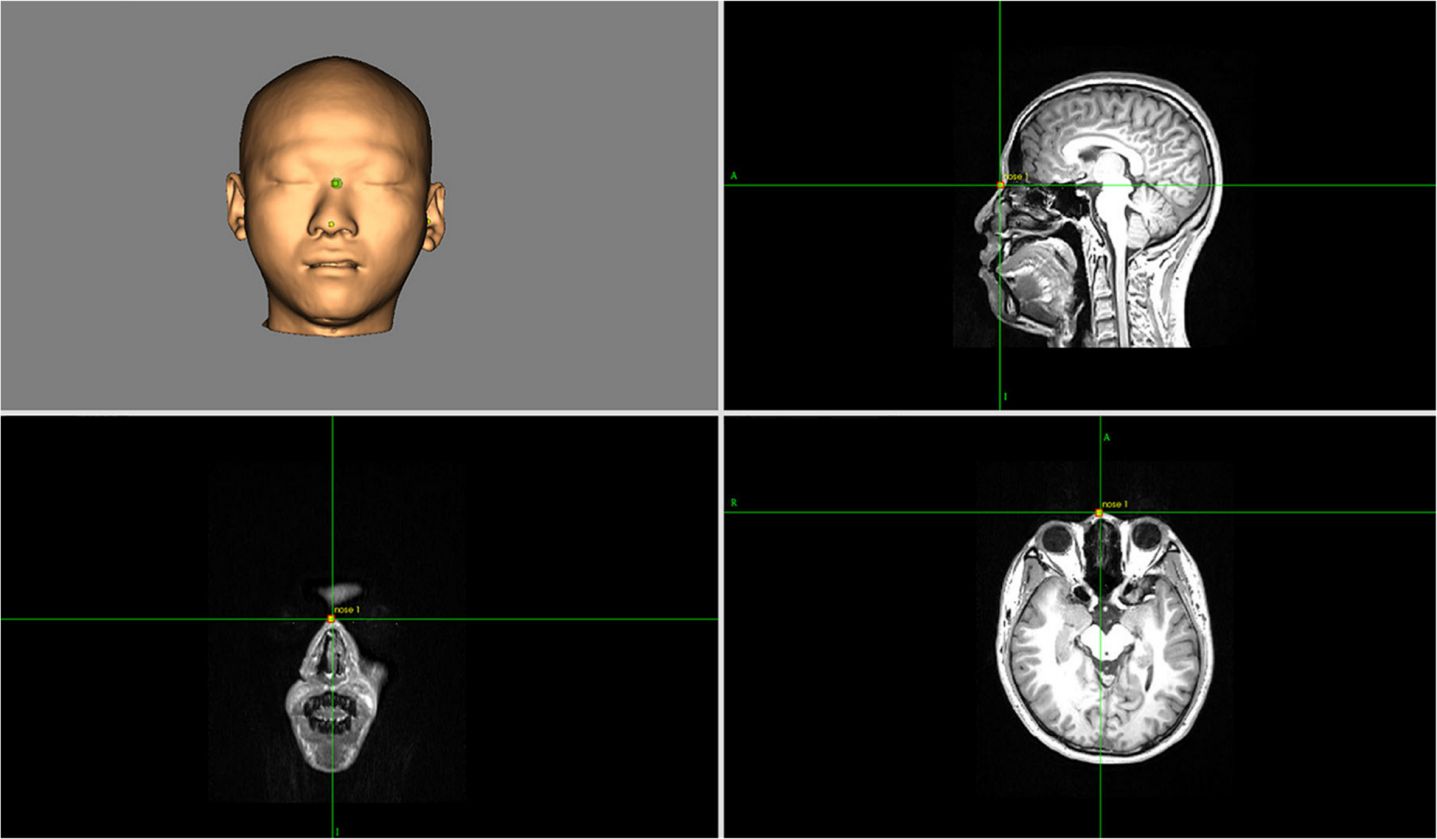
Three-dimensional (3-D) brain reconstruction

**Fig. 4:**
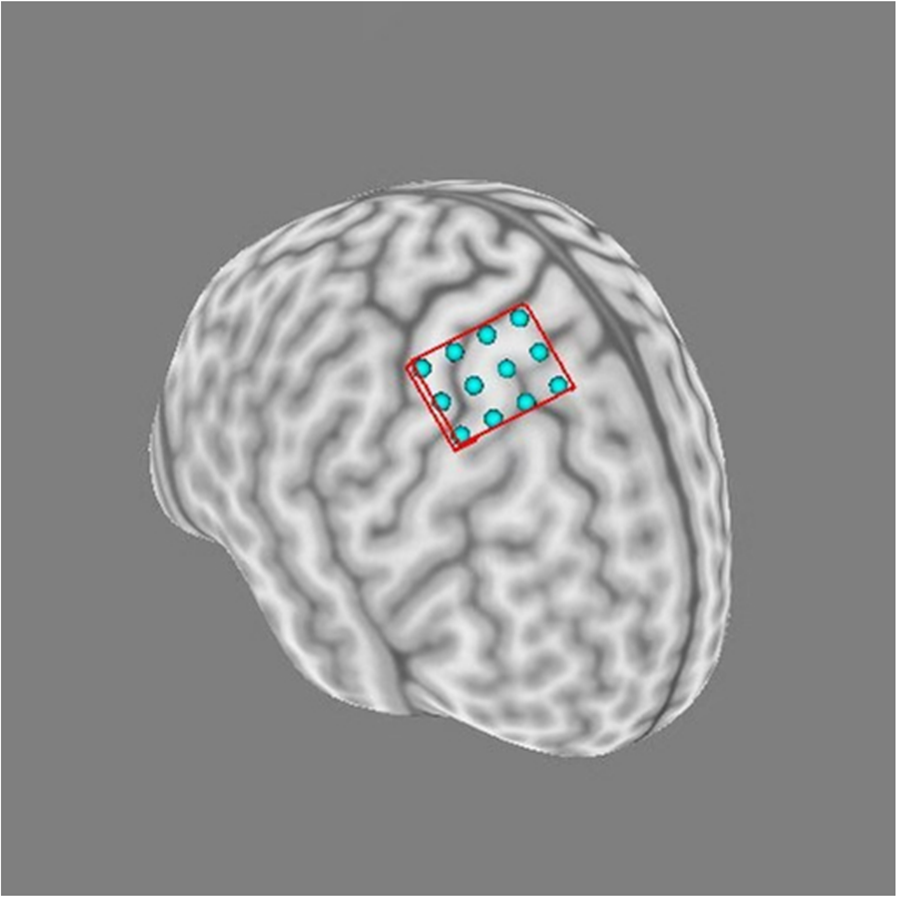
Targets with a 4×3 square grid built on the M1 region on the 3-D brain

All participants will undergo MRI (3.0 T) at Guangdong 999 Brain Hospital with an MRI scanner (Signa EXCITE 3.0 T HD, IGE, USA), and an MRI T1 file will be imported into the neuroimaging navigation system (Brainsight® 2.3.3.dmg) to reconstruct a 3-D brain (Fig. 3). Targets with a 4×3 square grid will be built on the M1 region on the 3-D brain (Fig. 4). The neuroimaging navigation system will be used to follow the stimulator coil of the TMS in real time.

### Transcranial magnetic stimulation

TMS will be performed by using a figure-eight stimulator coil before and after the intervention. The stimulator coil will be moved over the left and right M1 in order to identify the TMS hotspot [26] among targets based on the 4×3 square grid on the M1 region on the 3-D brain.

### Electromyography (EMG) measurement

RMTs and MEPs induced by TMS will be recorded from bilateral thenar muscles. Surface electrodes will be used to record the RMTs and MEPs of the thenar muscles and placed on the skin over the thenar muscles after skin disinfection. The surface recording electrodes will be placed bilaterally on each side of the thenar muscles, and the reference electrodes will be placed directly next to them (1 cm). Two ground wire disc electrodes will be placed on the upper edge of radius styloid process to reduce interference to the EMG signal. All electrodes will be checked every 15 min to ensure that they are in good contact with the skin and underlying muscles.

### Interventions

#### For healthy participants

Firstly, after skin disinfection, the K Streitberger placebo [27], the validated and reliable single-blind acupuncture needles (Huatuo, Suzhou Medical Supply Factory Co, Ltd, Suzhou, China), will be placed on the skin of Quchi (LI11), Zusanli (ST36), Hegu (LI4), and Chongyang (ST42) of the left limb of all healthy participants separately. Secondly, four sterile acupuncture needles (size 0.30×25 mm and 0.30×40 mm; Huatuo, Suzhou Medical Supply Factory Co., Ltd, Suzhou, China) will be inserted separately through the K Streitberger placebo of the left limb. For healthy participants in electroacupuncture groups, the acupuncture needles will be inserted approximately 15–30 mm into the skin and connected to an acupuncture point nerve stimulator (HANS-200A). The electroacupuncture frequency will be 2 Hz, 50 Hz, and 100 Hz, the intensity will be set to the maximum-tolerated intensity for each participant, and the time will sustain 20 min. Based on the above operation, the participants in electroacupuncture groups feel the Deqi sensation (a sensation of soreness, distention, numbness, or radiating that indicates effective needling). Besides, for healthy participants in the sham electroacupuncture group, the difference is that the acupuncture needles will be placed on the skin through the K Streitberger placebo without inserting into the skin and the K Streitberger placebo will be connected to the same stimulator (HANS-200A) without electricity output. Thus, the participants in the sham electroacupuncture group will not feel the Deqi sensation. All healthy participants will only receive one electroacupuncture or sham electroacupuncture intervention instantaneously.

#### For patients

All patients with post-stroke hemiplegia will receive conventional treatment according to the *Guidelines for the diagnosis and treatment of acute ischemic stroke in China* 2018 [28], which includes active control of blood pressure and blood sugar level, nutritional support, administration of the neurotrophic drug citicoline sodium 0.1~0.2 g, three times a day, orally; and statin treatment if considered necessary by the clinician. The patients will receive the anticoagulant aspirin 0.15~0.30 g, once a day, orally, and patients who cannot tolerate aspirin will receive clopidogrel instead. The patients will also receive basic rehabilitation training, including physical therapy, occupational therapy, and additional training as appropriate, e.g., weight loss support training or isokinetic muscle training. In addition, patients who suffered from the right side of the affected limb due to post-stroke hemiplegia in the opposing needling group will receive electroacupuncture on the left side of the unaffected limb in total 10 times (once per day for 5 consecutive days followed by a 2-day break altogether 2 weeks.) The acupoints and the operation method of electroacupuncture were described as the healthy participants in electroacupuncture groups. The electroacupuncture frequency will be the optimal frequency acquired from the healthy participants in electroacupuncture groups and the intensity will be set to the maximum-tolerated intensity for each patient and the time will sustain 20 min. Besides, the patients in the conventional treatment group will receive sham opposing needling intervention on the left side of the unaffected limb in total 10 times. The course of treatment will be the same as the patients in the opposing needling group. The operation method of sham electroacupuncture was also described as the healthy participants in sham electroacupuncture groups.

To maintain study fidelity, all researchers will receive training on the study methods, techniques, and study monitoring. All research staff will be tested after training to ensure they can consistently replicate the protocol.

### Outcome measures

#### Primary outcome measure

The primary outcome measure will be the per cent change in the RMTs of the thenar muscles at baseline and after the intervention. The RMTs of the thenar muscles will be delivered by a TMS pulse every 30 s bilaterally to the left or right M1, respectively, for 10 pulses in the fully relaxed state. The MEP of the thenar muscles should be greater than or equal to 50 μV for at least half (5/10) of the TMS pulses. The RMT is defined as the minimum intensity that is needed to elicit the MEP ≥ 50 μV in at least 50% of the pulses.

### Secondary outcome measures

The secondary outcome measures will be the amplitude (μV) and latency (ms) of the MEPs of the thenar muscles at baseline and after the intervention. The MEPs will be induced by a single TMS pulse as a proxy for the TMS-evoked potential in this study. In practical terms, the RMTs, which is the minimum intensity of the TMS from each participant, will be detected and recorded first. Then 110% of the RMTs value will be used as the relative stimulation intensity of the TMS, which will be calculated by adding 10 percentage points of the basic RMTs value to detect the excitability of the bilateral M1 every 30 s for three TMS pulses in this study [29].

### Safety and adverse events

To ensure safety and protect the rights of the participants, they will be assessed before and after the treatment and instructed to report any abnormal reactions or uncomfortable sensations to the researchers. Treatment-related adverse events, including bleeding, haematoma, pain, or vegetative symptoms, will be treated and closely monitored by the researchers [30]. However, for serious adverse effects such as hearing impairment or seizures, the trial will be immediately stopped and necessary medical treatments will be applied. These serious adverse events will be recorded and subsequently monitored by the China Clinical Trial Registration Ethics Committee.

### Data collection and management

The demographic information for each participant, including the participant’s age, sex, height, weight, dominant hand, medical history, outcome data, and reason for attrition, will be recorded in the case record form. Data will be labelled by a unique numeric identifier and recorded by the investigator. Upon completion of the trial, the electronic data will be examined twice by two independent data managers, and value pairs will then be compared for discordances, followed by resolution of discordances by referral to the original data. In addition, all participant’s data will be encoded in the ResMan Research Manager, which is a public clinical trial data acquisition and management system that was established by the data monitoring committee of the Chinese Clinical Trial Registry. The China Clinical Trial Registration Ethics Committee will also audit the study randomly as an independent body. The data monitoring committee will be responsible for data monitoring to evaluate safety issues every 6 months and monitor attrition rates; adverse events will be monitored quarterly, but special attention will be given to events requiring medical or surgical intervention that require hospitalisation and/or prevention of death and long-term disability.

The principal investigator, the co-investigators, and the acupuncturists administering the intervention are all experienced acupuncturists who can adroitly handle an acupuncture emergency. To maintain study quality, the researchers, including the acupuncturist and research assistants, will receive specialised training with a test-retest of all trial methods, study techniques, and monitoring methods. The outcome assessment and data analysis study personnel will be blinded to the study procedures. Participation will be terminated upon participant request or withdrawal of consent. Investigators may also exclude participants from the study to ensure their safety.

### Sample size calculation

The sample size for the 2-Hz, 50-Hz, 100-Hz, and sham electroacupuncture groups was 20, which was calculated according to the following statistical formula: n = ${\upvarphi}^2\Big(\sum {\mathrm{S}}_{\mathrm{i}}^2/$g)/[$\sum {\left({\overline{\mathrm{X}}}_{\mathrm{i}}-\overline{\mathrm{X}}\right)}^2/\left(\mathrm{g}-1\right)\Big]$, where g represents the number of groups and group allocation ratios are equal. Based on the preliminary experiment, the average RMT reductions in each group were expected to be − 4.44, − 3.33, − 1.75, and − 0.91, with standard deviations of 3.69, 2.89, 3.40, and 1.92, respectively. The *P*-value for the two-sided test was 0.05, and the test power was 80%. Thus, using PASS 15 software, the optimal sample size was 72. Considering a 10% dropout rate, a total of at least 80 participants would have to be included, with 20 participants included in each group.

For patients with post-stroke hemiplegia in the opposing needling or conventional treatment groups, the sample size was 20 based on the statistical formula *n* = ${\left[\frac{\left({Z}_{\propto }+{Z}_{\beta}\right)\upsigma}{\updelta}\right]}^2$. The main primary outcome measure is RMT. *δ* is the RMT of the thenar muscles, and according to our previous trials, the minimum clinically significant RMT value was estimated to be 3.57 [24]. Thus, *δ* was estimated to be 3.57. ∝is the significance level; *β* is the power of the test. In this study, ∝= 0.05, *Z*_∝_= 1.96, *β* = 0.80 (80%), and *Z*_*β*_ = 0.84, while σ was estimated at 3.67. With the PASS 15 software, the optimal sample size of the opposing needling and conventional treatment groups was calculated as 18 cases. Considering an overall dropout rate of 10%, including cases lost to follow-up and patients refusing to follow-up, at least 20 participants would be required in the treatment and control groups. Thus, at least 40 patients with post-stroke hemiplegia would have to be recruited for the study.

### Statistical analysis

Statistical analysis of the RMTs and MEPs will be performed with Statistical Package for Social Science (SPSS) software for Windows, version 17.0 (SPSS Inc., Chicago, IL, USA). D’Agostino’s test for normally distributed variables and Levene’s test for homogeneity of variance will be applied. For healthy participants, if measurement data are normally distributed, then Bonferroni’s test will be used to analyse between-group differences for each participant pre- and post-electroacupuncture or sham electroacupuncture. Otherwise, Kruskal-Wallis’ H test for a nonparametric distribution will be used. The level of significance is set at 5% for all comparisons. For patients with post-stroke hemiplegia, if the measurement data are normally distributed, then an independent *t* test will be used to analyse between-group differences for each participant pre- and post-electroacupuncture or sham electroacupuncture. Otherwise, the Wilcoxon rank-sum test for a nonparametric distribution will be used. The level of significance will be set at 5% for all comparisons.

### Patient and public involvement

Patients and the public are not involved in the design or conduct of the study or the outcome measures, and the burden of intervention will not be assessed by the trial participants. Individuals involved in the consultations related to the trial design will not be included as trial participants.

## Discussion

### Acupuncture point selection

Previous studies suggested that the most commonly used meridians in clinical treatment of post-stroke hemiplegia are the large intestine meridian of hand-Yangming (LI), stomach meridian of foot-Yangming (ST), and gallbladder meridian of foot-Shaoyang (GB); the most commonly used acupoints are LI11, ST36, LI4, etc. [31]. According to Traditional Chinese Medicine theory, hemiplegia after stroke belongs to the category of flaccidity syndromes, and *The Huangdi’s Classic of Internal Medicine* noted that treatment for flaccidity is aimed at the Yangming meridian. The Yuan primary point is an acupuncture point that represents this meridian and also one of the most commonly used acupuncture points. Therefore, we will select the two of the most commonly used acupuncture points from LI and ST, LI11 and ST36, and choose the Yuan primary points of LI and ST, LI4 and ST42.

### Effect of different frequencies of electroacupuncture on regulation of stroke

Compared to traditional acupuncture, electroacupuncture has the advantage of high reproducibility of the therapeutic effects, the precision of stimulation parameters and a significant reduction in labour. This new acupuncture therapy is becoming more and more popular. Different frequencies of electroacupuncture could produce different effects. For neurological reconstruction, electroacupuncture could promote the expression of NGF and BDNF in the hippocampal CA3 region of cerebral ischemic rats, and the 50-Hz electroacupuncture effect was more obvious [32]. For cerebral blood flow, the electroacupuncture in both 2 and 15 Hz groups induced the increase of cerebral blood flow in rats [33]. Besides, electroacupuncture could improve local cerebral blood perfusion and brain cell functions, and the effect of 2/15 Hz and 100 Hz were better [34]. For motor functional recovery, low-frequency electroacupuncture could promote recovery of motor function after focal cerebral ischemic injury in rats [35]. Electroacupuncture in the 2-Hz group had an obvious effect on dysphagia after stroke in mice [36]. However, there are few researches on the effect of different frequencies of electroacupuncture or opposing needling on motor cortex excitability in healthy participants or in patients with post-stroke hemiplegia. The effect of different frequencies of electroacupuncture on bilateral motor cortex excitability should be studied further. Due to even at the maximum stimulation in TMS, the affected hemispheres of some patients with post-stroke hemiplegia often fail to induce MEPs. Thus, we design this study to explore the effect of different frequencies of electroacupuncture on bilateral motor cortex excitability in healthy participants and then acquire the optimal frequency of electroacupuncture for motor cortex excitability. Lastly, we will apply this frequency of electroacupuncture to patients with ischemic stroke to explore its effect on motor cortex excitability.

### Influence of the corpus callosum on excitability/inhibition of unaffected and affected hemispheres

The connection between the brain and the limbs is bilateral; 80% of the nerve fibres are crossed, which dominate the movement of the contralateral limb, while a small part is not crossed, which directly descends to form the anterior bundle of the cortical spinal cord and dominates the movement on the same side. The corpus callosum, which connects the two hemispheres of the brain, is the largest white matter body in the mammalian brain and contains more than 200 million myelinated axon fibres [37]. The corpus callosum is responsible for information transmission and functional integration between the two hemispheres [38]. Researchers who supported the corpus callosum excitatory model believed that the corpus callosum transmits excitatory information between the two hemispheres and that it can activate the unstimulated cerebral hemisphere, further promoting an exchange of information between the two hemispheres, and facilitating the integration of the functions of the two hemispheres. For patients after stroke, some TMS studies have suggested that the activity in the residual networks in the unaffected hemisphere may contribute to functional recovery after stroke by substituting for the functions lost by the damaged area. However, the interhemispheric competition model suggested exactly the opposite. This model assumes the presence of a mutual, balanced inhibition between the hemispheres in the healthy brain. Damage to the hemisphere by a stroke disrupts this balance, and inhibition of the unaffected hemisphere by the affected hemisphere is reduced, resulting in increasing inhibition of the affected hemisphere by the unaffected hemisphere [39]. One study suggested that patients exhibit over activity in their unaffected M1 after stroke and show high levels of neural excitability, while the affected M1 exhibits a low excitatory level [40]. Another study showed that skin anaesthesia in the unaffected hands of stroke patients can lead to improved behaviour of the affected hands, which supported the interhemispheric competition model [41]. However, the two opposing theories mentioned above may be oversimplified or even incorrect. To better explain the relationship between the balance and functional recovery of the two hemispheres in pathological conditions, Giovanni Di Pino proposed a bimodal balance–recovery model. This model links interhemispheric balancing and functional recovery to the structural reserve spared by the lesion. The proposed ‘structural reserve’, which was described the remaining functional motor output, determined the amount of recovery limited by interhemispheric competition and contributed to the recovery in an individual patient [39]. The rebalancing of interhemispheric competition may play an important role in the process of motor recovery. Previous studies have extensively studied the effects of the affected cerebral hemisphere to better understand changes in motor network activity after stroke, but few studies have examined the effects of the unaffected brain hemispheres. The motor recovery after ischemic stroke in the primary motor cortex may occur partly through training-enhanced reorganisation in undamaged premotor areas, enabled by reductions in cortical inhibition [42]. One study suggested that the restoration of function after stroke is associated with biphasic recruitment of peri- and contralesional functional fields in the brain [43], that the connectivity patterns in the unaffected hemisphere more accurately reflect the behavioural conditions than those in the affected hemisphere [44], and that in comparison with the unaffected hemisphere, the affected hemisphere shows reduced connections from the superior parietal cortex to M1 and supplementary motor cortex [45]. An acupuncture study showed that acupuncture at Yanglingquan (GB34) on the affected limb can reduce compensatory activity in the healthy side of the brain. These studies have shown that the unaffected hemisphere plays an important role in stroke recovery.

### Study on the mechanism of opposing needling in the treatment of patients with post-stroke hemiplegia

Opposing needling has obvious curative effects in the treatment of post-stroke hemiplegia, however, the mechanisms underlying its therapeutic effects remain unclear. In terms of neuroanatomy, some scholars believed that the cerebral cortex, the non-specific projection system of the thalamus, the brainstem network structure, and the spinal cord are the neuroanatomical basis for the effect of opposing needling [46]. In terms of the signal transduction pathway, a study suggested that opposing needling markedly protected the brain against transient cerebral ischemic injury and this effect was probably mediated by the activation of the GABAB/cAMP/PKA/CREB signal transduction pathway [24]. In terms of rehabilitation kinesiology, opposing needling was based on the active use of joint response by stimulating the unaffected muscle to induce contraction of the affected muscle, to inhibit spasm and antagonism of muscle, and further to improve motor function [47].

### Biomarkers for predicting upper limb motor function after stroke

Upper limb motor function is difficult to predict in stroke survivors. One clinical study pointed out that the FMA may be used as a tool to define motor recovery and guide the choice for therapy, and patients who fit that model showed a fixed proportional upper extremity motor recovery of about 78%. However, this proportional recovery model was seriously challenged by some severe types of stroke [48]. Researchers are still struggling to explain these different patterns of stroke recovery. Thus, the development and implementation of biomarkers in stroke recovery research are difficult. During the acute phase after stroke, even at the maximum stimulation in TMS, the affected hemisphere often fails to induce MEPs. In patients with preserved MEPs, the MEPs are smaller and motor thresholds are generally higher than those recorded from unaffected hemispheres or healthy individuals. Within the first few months, MEPs may reappear again and gradually increase, and the RMTs of the affected hemisphere tends to decrease [49–53]. Some studies have reported that TMS measures correlate with the long-term functional outcome, such as corticospinal tract integrity evaluated early after stroke [50], and that improvements in TMS measures reflect improvement in corticospinal integrity during the first few months of recovery [54]. Stinear proposed the ‘PREP algorithm’, an important component of which is the presence or absence of MEPs in arm muscles within the first week after stroke, which, in combination with the TMS response, clinical upper limb strength measures, and MRI measures of corticospinal tract asymmetry, can predict the recovery prospects for individual patients [31, 55–57]. For the RMTs of the unaffected hemisphere, the meta-analysis by McDonnell found no differences compared to healthy controls, regardless of the stage of stroke [56]. Catano found no obvious correlation between RMTs changes and lesion location [51]. In contrast, Charlotte Rosso conducted a systematic review of relevant studies and found a correlation between RMTs and upper limb motor function after stroke, indicating that RMTs may be regarded as a potential biomarker of post-stroke upper limb motor function [57]. Thus, it is possible to regard MEPs and RMTs as suitable biomarkers for predicting post-stroke upper limb function.

## Trial status

The first day of recruitment was 1 January 2020. Last recruitment of participants will be planned for February 2022. Current protocol version is version 2.0 dated 13 December 2019.

## Supplementary Information

**Additional file 1.** SPIRIT 2013 checklist: recommended items to address in a clinical trial protocol and related documents.

## Data Availability

The datasets used and/or analysed during the current study will be available from the corresponding author on reasonable request after the publication of the results.

## Abbreviations

M1: Primary motor cortex
TMS: Transcranial magnetic stimulation
RMTs: Resting motor thresholds
MEPs: Motor-evoked potentials
tPA: Tissue plasminogen activator
RCT: Randomised, controlled trial
MMSE: Mini-Mental State Examination
BMI: Body mass index
FMA: Fugl-Meyer assessment
MRI: Magnetic resonance imaging
NIHSS: National Institutes of Health Stroke Scale
EMG: Electromyography
SPSS: Statistical Package for Social Science
LI: Large intestine meridian of hand-Yangming
ST: Stomach meridian of foot-Yangming
GB: Gallbladder meridian of foot-Shaoyang
